# The Role of Belimumab in Systemic Lupus Erythematosis: A Systematic Review

**DOI:** 10.7759/cureus.25887

**Published:** 2022-06-13

**Authors:** Ashna Joy, Abilash Muralidharan, Marwa Alfaraj, Darshan Shantharam, Akhila Sai Sree Cherukuri, Arun Muthukumar

**Affiliations:** 1 Internal Medicine, AP Varkey Mission Hospital, Ernakulam, IND; 2 Internal Medicine, Kiruba Hospital, Coimbatore, IND; 3 Internal Medicine, LaSante Health Center, New York, USA; 4 Internal Medicine, Yenepoya University, Mangalore, IND; 5 Anaesthesiology, Calcutta National Medical College & Hospital (CNMCH), Kolkata, IND

**Keywords:** monoclonal antibody, systemic lupus erythematosus, blys receptor, rheumatology & autoimmune diseases, belimumab

## Abstract

Systemic lupus erythematosus (SLE) is a chronic autoimmune disease involving multiple systems with a range of clinical presentations caused by the production of antibodies, activation of complements, and deposition of immune complexes. The exact cause of SLE is still unknown. The effectiveness of traditional treatment methods for SLE is very little. Nowadays, resistance to conventional therapy, steroids, and immunosuppressants is common among SLE patients. Patients with refractory disease and nephritis generally have severe drug-induced toxicity which contributes to organ dysfunction, despite available therapies. Different biologic agents and therapeutic antibodies have become an alternative and have been under experiment in clinical trials, enrolling patients whose disease is inadequately controlled by conventional treatment. Belimumab is the only targeted therapy approved for SLE treatment. This systematic review discusses one such biological agent for treating systemic lupus erythematosus, namely, belimumab.

The systematic review was conducted in accordance with the Preferred Reporting Items for Systematic Reviews and Meta-Analyses (PRISMA) guidelines. Studies included randomized clinical trials (RCTs) from 2005 to 2021 on adult SLE. patients treated with monoclonal antibodies to assess the efficacy and safety. Methodological quality was assessed using PubMed, PMC, the Cochrane Risk of Bias tool, and the QUality In Prognosis Studies Tool (QUIPS) for RCTs. Two independent reviewers performed an electronic search on MEDLINE, Cochrane Library, SCIELO, Scopus, and ResearchGate.

Based on a systematic review of articles we found that belimumab appears to be efficacious and generally well-tolerated in the treatment of SLE as compared to other drugs. The long-term use of belimumab combined with standard therapy showed a low incidence of organ damage. A lower incidence of organ damage was seen after initiating treatment in patients with a high risk for organ dysfunction. Patients who test for antinuclear antibody or anti-dsDNA-positive SLE, with moderate symptoms in the skin and musculoskeletal systems despite immunosuppressants, are treated with belimumab as an adjunct therapy. Patients with severe lupus nephritis or active CNS lupus cannot be treated with belimumab. Belimumab is effective in most races, as a clinical trial done in North-East Asia showed improvement in SLE symptoms and decreased dependence on prednisone. Belimumab also decreased disease activity and severe flares. Belimumab had greater efficacy in children.

## Introduction and background

Systemic lupus erythematosus (SLE) is a chronic autoimmune disease involving multiple systems with a range of clinical presentations caused by the production of antibodies, activation of complements, and deposition of immune complexes. The exact cause of SLE is still unknown [1]. Still, epidemiologic evidence exists for the associations of silica, cigarette smoking, oral contraceptives, postmenopausal hormone therapy, and endometriosis with SLE incidence [2]. Recent studies have also provided robust evidence of the association between alcohol consumption and decreased SLE risk [3]. The immunology of SLE involves dysregulation of the immune response, including innate immunity and adaptive immunity [4]. B lymphocyte has an essential role in the adaptive immune response of SLE, which involves the production of autoantibodies, presentation of autoantigens, and activation of autoreactive T cells. Furthermore, T lymphocyte plays a role through the co-stimulator-mediated signaling pathway and cytokines secreted by subsets of T cells [5]. The heterogeneous nature of lupus makes it challenging to identify sensitive and specific markers for its diagnosis and monitoring [6].

The effectiveness of traditional treatment methods for SLE is very little. Nowadays, resistance to conventional therapy, steroids, and immunosuppressants is common among SLE patients [7]. Patients with refractory disease and nephritis generally have severe drug-induced toxicity, which contributes to organ dysfunction, despite the available traditional therapies [8]. Different biologic agents and therapeutic antibodies have become an alternative and have been under experiment in clinical trials enrolling patients whose disease is inadequately controlled by conventional treatment regimens [9]. Belimumab is the only targeted therapy approved for SLE treatment, as treatment with many biologic agents has given unsatisfactory results [10]. Several novel biologic agents targeting B cells, T cells, or cytokines are undergoing clinical trials for the treatment of SLE [11]. With a deepening understanding of pathogenesis, targeted therapy has become a more promising treatment, especially for patients who are resistant to conventional therapies [12]. Patients have started developing tolerance toward conventional therapies like steroids and immunosuppressants [13]. At present, moderate and severe SLE are treated with biological agents [14]. In cases where the immunosuppressive agents and hormone therapy are ineffective, biological agents control the disease levels [15].

This systematic review discusses one such biological agent for treating systemic lupus erythematosus, namely, belimumab.

## Review

Methods

The systematic review was conducted in accordance with the Preferred Reporting Items for Systematic Reviews and Meta-Analyses (PRISMA) guidelines.

Screening of Articles

Inclusion criteria included papers from 15 years, published in English, an adult population whose age ranged from 18 to 65 years, female, and randomized clinical trials (RCTs).

Exclusion criteria were pediatric population, geriatric population, unpublished papers, and gray literature.

Search Strategy

Studies included RCTs from 2005 to 2021 on adult SLE patients treated with monoclonal antibodies to assess the efficacy and safety of clinical responses like arthritis, mucocutaneous and renal response, relapse, and side effects. RCTs included for methodological quality were assessed using PubMed, PubMed Central, the Cochrane Risk of Bias tool, and the QUality In Prognosis Studies Tool (QUIPS) for RCTs. The quality of subgroup analyses testing predictors of differential treatment response was also evaluated. The best evidence synthesis was performed using the Grading of Recommendations Assessment, Development, and Evaluation (GRADE) framework.

Two independent reviewers performed an electronic search on MEDLINE, Cochrane Library, SCIELO, Scopus, and ResearchGate. The following descriptors were used: SLE, randomized clinical trials, etanercept, adalimumab, golimumab, certolizumab pegol, rituximab, ocrelizumab, epratuzumab, abetimus, edratide, belimumab, atacicept, abatacept, efalizumab, sirolimus, infliximab, sifalimumab, rontalizumab, anakinra, tocilizumab, and eculizumab. Boolean operators, such as ‘‘AND’’ and ‘‘OR’’, were also used in the electronic search. The search was concluded on the 10th of April, 2022.

Results

**Figure 1:**
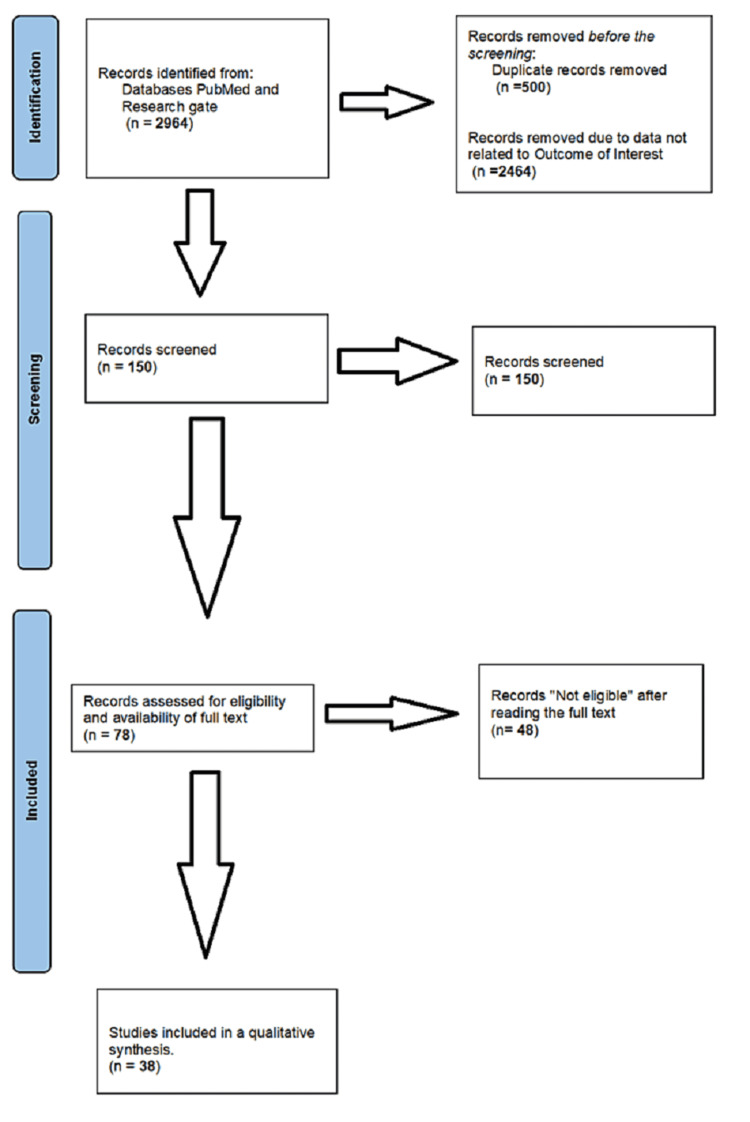
Search Strategy and Screening of Articles

This systematic review initially identified 2964 articles; 500 duplicate records were deleted and another 2464 were removed since their outcome was not of interest. Due to the difference in exclusion criteria, another 72 records were not considered. The remaining 78 articles were further filtered after reading the complete text, and 38 articles were eventually considered in this systematic review (Figure 1).

The total number of patients included in trials involved in this systematic review is 6598. Even though most studies had belimumab as the primary intervention compared to control, other studies included other monoclonal antibodies such as ustekinumab, anifrolumab, epratuzumab, AMG, sifalimumab, and other monoclonal antibodies. The upper limit of age was not available in all the studies we discuss, but the lower age limit is five years.

Different indicators were used to assess the efficacy of most interventions with some similarities, including an improvement of several SLE scores, making a unified systematic review difficult. Therefore, we will discuss several studies separately, especially those using other monoclonal antibodies than belimumab.

Discussion

Goals of Treatment of SLE

The objectives of treatment for lupus are to maintain a very low disease activity and avoid triggers known to cause disease flare-ups, prevent damage to organs from disease activity, and decrease the secondary complications of lupus and its treatment [16]. Initiating treatment early and having a partnership with the patient toward the above-mentioned goals, is essential. This includes avoidance of known triggers that cause flares, the need for sun protection, avoiding the use of prednisone for maintenance therapy, and controlling active disease with immunosuppression or biologics [17].

Drugs Commonly Used to Treat SLE

a) Immunomodulators: The various immunomodulatory drugs used in SLE are:

Hydroxychloroquine is commonly used in every patient, as it modulates the immune response by inhibiting B cell receptors, TLR signaling, intracellular TLR-3, and -7 activation, which plays a very important role in nucleic acid-sensing. It also interferes with the lysosomal pH and MHC-antigen binding, which is the reason for it being the cornerstone of treatment for lupus [18].

Vitamin D has to be used as a supplement in all SLE patients because of its modulatory role in the immune system and anti-fibrotic effects [19].

Dehydroepiandrosterone (DHEA) is used in the treatment of SLE because of its regulatory effects on proinflammatory cytokines like interleukin (IL)-2, IL-1, IL-6, and tumor necrosis factor-alpha (TNF-a). It also causes a reduced antibody production in mice [20].

Corticosteroids are used to rapidly ablate the autoimmune response in organ-threatening manifestations such as nephritis, vasculitis, central nervous system lupus, myocarditis, or alveolitis. In lupus patients, 80% of organ damage after diagnosis is attributable to prednisone use. Lower doses can increase the risk of cataracts, osteoporosis, fractures, and coronary artery disease. Doses above 6 mg increase the risk of organ damage by 50% and doses of 10 to 20 mg daily increase the risk of cardiovascular events [21].

b) Cytotoxic drugs: The various cytotoxic drugs used in SLE are:

Cyclophosphamide is an alkylating agent that acts by depleting T and B cells and producing antibodies. It is widely used to induce and maintain lupus nephritis and central nervous system lupus. It has been replaced by immunosuppressive medications that are less toxic [22].

Azathioprine has been commonly used in renal and extrarenal lupus. Two randomized studies that compared treatment with azathioprine to corticosteroids alone showed a reduction in mortality and flares, especially in patients with severe renal or central nervous system disease. Given its inferiority to cyclophosphamide, its use has decreased over time [23].

Methotrexate is an antimetabolite used in the treatment of SLE, as it interferes with DNA synthesis, repair, and replication by irreversibly binding to dihydrofolate reductase. Methotrexate reduces disease activity in SLE, especially in joint and skin diseases. It also facilitated anti-dsDNA and complement levels. Methotrexate showed modest steroid-sparing activity in a randomized controlled trial [24].

Mycophenolate is used in the treatment because it acts by preferentially depleting guanosine nucleotides in T and B cells, which inhibits their proliferation. Mycophenolate is effective for the induction and maintenance of lupus nephritis [25].

Calcineurin inhibitors are used in the treatment of SLE, as it targets T cells by preventing the inhibition of calcineurin. Multiple small RCTs showed that the induction of lupus nephritis cyclosporine or tacrolimus is equally effective as cyclophosphamide or mycophenolate [26].

c) Monoclonal antibodies: The various monoclonal antibodies used in SLE are:

Rituximab is used in the treatment of SLE, as it is an anti-CD20 monoclonal antibody that leads to the depletion of peripheral B cells [27]. Several observational studies showed that rituximab was beneficial in renal and non-renal lupus [28]. There were two large randomized placebo-controlled trials with rituximab in renal and non-renal lupus that failed to meet their primary outcomes. In the EXPLORER non-renal trial with rituximab, the primary endpoint, which was the proportion of patients achieving complete or partial remission based on the BILAG score, was not met. B-cell depletion was seen in about 90% of SLE patients treated with rituximab. Rituximab did reduce anti-dsDNA titers and improved C3 and C4 levels [29]. The LUNAR renal trial showed that rituximab did reduce proteinuria better than mycophenolate alone at 18 months. Hence, the above-mentioned clinical trials clearly show that the use of rituximab in the treatment of SLE is still controversial [30].

Belimumab Effects on SLE Disease Activities

Belimumab mechanism of action in SLE: One of the pathogenic mechanisms of SLE is the overexpression of B Lymphocyte Stimulator (BLyS), which promotes B cell survival, especially autoreactive B cells. BLyS inhibition results in apoptosis of autoreactive B-cell. BLyS plays an important role in the pathogenesis of SLE. SLE patients generally have elevated circulating BLyS levels compared to healthy individuals. Higher levels of BLyS correlate with increased disease activity. Higher levels of BLyS also correlate with increased anti-double-stranded DNA (dsDNA) antibody titers. Therefore, the biological activity of BLyS has to be inhibited, which is necessary for the treatment of the disease. Belimumab acts by binding to soluble BLyS [31].

Belimumab is a complete human monoclonal antibody against BLyS, a B-cell survival factor. It was licensed in 2011 for the treatment of autoantibody-positive SLE. Belimumab plays a very important role in B cell survival and function, as it belongs to the TNF superfamily.

Belimumab is the first biological agent to be approved by the Food and Drug Administration (FDA) for SLE. Early multicenter phase III clinical trials have shown that the long-term use of high doses continuously improved serological indicators, reduced hormone dosage, and reduced the risk of severe recurrence in SLE [32].

The BLISS-52 and BLISS-76 Phase III trials successfully demonstrated that once-a-week therapy with belimumab (10 mg/kg) with standard therapy significantly decreased disease activity in SLE patients both clinically and statistically compared to placebo with standard treatment. Both the clinical trials mentioned here met their primary outcomes in SLE [33].

Belimumab Effects on Lupus Nephritis

The design of the clinical trials mentioned above did not involve treating a frequent cause of SLE-associated morbidity and mortality, which was lupus nephritis because of the heterogeneous definitions of treatment response, clinical presentation, and renal involvement, which made it practically impossible for a comparison of the results using a single outcome parameter. Based on emerging evidence, there seems to be a potential therapeutic role for agents that target BLyS.

However, most of these above-mentioned studies and clinical trials defined the clinical response in renal flare and renal remission rates, which may gradually reduce the risk of proteinuria. Despite the limitations of the studies included, the data available seems to be promising by providing preliminary support for targeting BLyS, which will induce or maintain a renal response [34].

Recently, a randomized, double-blind, placebo-controlled phase 3 trial of intravenous belimumab in patients with active lupus nephritis, which was called the BLISS-LN trial showed that patients with lupus nephritis, who received belimumab plus standard therapy (MMF or AZA) had a better renal response when compared to those who received standard therapy alone.

As there is no data available on whether belimumab plays a role in patients who failed induction therapy or relapse, it is still not yet recommended as the only primary treatment for lupus nephritis [35].

Belimumab Effects on Serological Markers of SLE

Treatment with belimumab resulted in a 63-71% reduction of naive, activated, and plasmacytoid CD20+ B cells and a 29.4% reduction in anti-dsDNA titers (P = 0.0017) by Week 52. The adverse and severe events rates were similar in the belimumab and placebo groups.

Belimumab was biologically active and well-tolerated. The effect of Bbelimumab on reducing SLE disease activity or flares was not significant. However, serologically active SLE patients responded significantly better to belimumab and standard therapy than standard therapy alone [36].

SLE patients who were treated with Bbelimumab experienced a significant and sustained reduction in immunoglobulin G (IgG), autoantibodies, and an improvement in C3/C4 levels. This resulted in positive-to-negative conversion rates for IgG anti-dsDNA, anti-Sm antibodies, anticardiolipin antibodies, and anti-ribosomal P autoantibodies. This also led to the normalization of hypergammaglobulinemia and low C3/C4 levels. There was a significant decrease in naive and activated B cells and plasma cells. There was no decrease in memory B cells and T cell population.

Belimumab causes a normalization of serologic activity and reduces BLyS-dependent B cell subsets in SLE patients who are serologically and clinically active. Better treatment response to belimumab is predicted by greater serological activity.

Belimumab Effects on the Clinically Significant Disease Activities of SLE

A post hoc analysis showed that there were significant reductions in SLE disease activity. There was a decrease in the risk of severe flares in patients who were anti-dsDNA positive and had low C3/C4 levels at baseline treated with belimumab 10 mg/kg (P≤0.01). The serum levels of C3 and anti-dsDNA were back to the normal range by eight weeks, which was irrespective of therapy. This predicted a reduced risk of severe flares over 52 weeks [37].

A pooled post hoc analysis of phase 3, randomized, placebo-controlled BLISS trials (1684 patients with active systemic lupus erythematosus) was performed to evaluate the effect of belimumab on renal parameters in patients with renal involvement at baseline. The analysis was also done to explore whether belimumab provided additional renal benefit to patients receiving mycophenolate mofetil at baseline.

In the clinical trial that treated patients with SLE with belimumab for over 52 weeks, the rates of renal flare, renal remission, renal organ disease improvement (assessed by Safety of Estrogens in Lupus Erythematosus National Assessment - Systemic Lupus Erythematosus Disease Activity Index and the British Isles Lupus Assessment Group), proteinuria reduction, grade 3/4 proteinuria, and serologic activity was assessed. The results of the trial favored belimumab.

Among the 267 patients with renal involvement at baseline, a more remarkable renal organ disease improvement with belimumab was seen than in those receiving mycophenolate mofetil. The limitations of this analysis were the small patient population and the post-hoc nature of this pooled analysis. The results suggest that SLE patients with renal involvement benefited from belimumab therapy [38].

In the following table, we discuss and compare in detail various clinical trials done about Belimumab and other monoclonal antibodies used in the treatment of SLE (Table 1).

**Table 1:** Systematic Review of Clinical Trials Involving Monoclonal Antibodies Used for the Treatment of SLE SLE: systemic lupus erythematosus; SRI: SLE responder index; SELENA: safety of estrogens in lupus erythematosus national assessment; SLEDAI: systemic lupus erythematosus disease activity index; PRINTO: Paediatric Rheumatology INternational Trials Organisation; ACR: American College of Rheumatology; S.O.C.: standard of care; anti-dsDNA: anti-double-stranded DNA; P.G.A.: Physician's Global Assessment; A.E.s: adverse events; S.A.E.s: serious adverse events; BILAG: British Isles Lupus Assessment Group; BICLA: BILAG-based composite lupus assessment

S.NO	Authors	Study design	Inclusion criteria	Exclusion criteria	Intervention	Primary outcome	Secondary outcome	Conclusion
1.	Bruce IN, Urowitz M et al [1]	open-label, long-term, continuation studies	Have completed the HGS1006-C1056 protocol in the United States through Week 72 visit. Be able to receive the 1st dose of Belimumab for H.G.S. 1006-c1066 four weeks after the last dose in HGS1006-c1056.	They have developed any other medical disease or condition that has made the subject unsuitable for this study in the opinion of their physician.	Belimumab 1 mg/kg IV over one hour every 28 days	Change in S.D.I. from baseline at study years 5–6	S.D.I. subgroup analyses (baseline S.D.I. 0 or ≥ 1, baseline Safety of Estrogen in Lupus National Assessment-Systemic Lupus Erythematosus Disease Activity Index (SELENA-SLEDAI) ≤ 9 or ≥ 10), and time to first S.D.I. worsening.	There was a low incidence of organ damage accrual in patients with SLE who were treated with long-term Belimumab plus SoC. There was also a low accrual in patients who were at high risk with pre-existing organ damage which suggested a favorable effect.
2.	Brunner HI, Abud-Mendoza C et al [2]	Phase-2, a randomized, placebo-controlled, double-blind study	That included a study population between 5 and 17 years who had clinically active SLE disease, defined as Safety of Estrogens in Lupus Erythematosus National Assessment-SLE Disease Activity Index (SELENA-SLEDAI) score ≥6 at screening,^13^ fulfilled ≥4 of 11 American College of Rheumatology (A.C.R.) criteria for the classification of SLE and had an unequivocally double-positive test result for antinuclear antibody ≥1:80 and anti-double-stranded (ds) D.N.A.≥30 I.U./mL antibodies.	Active central nervous system SLE or severe acute lupus nephritis (L.N.), or use of Prednisone systemically (or equivalent) at a dose of >1.5 mg/kg/day, B cell-targeted therapy in one year.	belimumab at a dose of 10 mg/kg intravenous on the same day, 14th day, and 28th day, then every 28 days until Week 48	SLE Responder Index 4 (SRI4) response rate at Week 52, defined as ≥4-point reduction from baseline in SELENA-SLEDAI score, no worsening in Physician's Global Assessment of SLE. activity (P.G.A.), that is, P.G.A. increase <0.30 points from baseline, no new British Isles Lupus Assessment Group (BILAG) A organ domain score; and no two recent BILAG B organ domain scores compared with baseline.	The proportion of patients responding to therapy defined by PRINTO/ACR SLE criteria,^17–19^, which considers the percentage of changes from baseline of the five multidimensional core components (P.G.A. (scale 0 to 3), Parent Global Assessment of overall patient well-being (Parent-global, scale 0 to 10), SELENA-SLEDAI, Paediatric Quality of Life Inventory (PedsQL; physical-functioning domain, scale 0 to 100) and proteinuria. Improvement in PRINTO/ACR 30 alternative definition is the proportion of patients with ≥30% improvement in three of five cycle core response criteria and with ≤1 of the remaining worsening by >30%, and in PRINTO/ACR 50 as the proportion of patients with ≥50% improvement in any two of five cSLE core response criteria and ≤1 of the remaining worsening by >30%.	Belimumab had greater efficacy in children than placebo.
3.	Cesaroni M, Seridi L et al [3]	phase II randomized, placebo-controlled study	Patients were those who had a diagnosis of SLE. (in accordance with the Systemic Lupus International Collaborating Clinics classification criteria for at least three months before the first study drug administration	Not applicable	Intravenous infusion of ustekinumab (~6 mg/kg) followed by subcutaneous injections of 90 mg ustekinumab or placebo every eight weeks, with placebo crossover to 90 mg ustekinumab every eight weeks. These treatments were administered in addition to standard‐of‐care therapy.	Efficacy was assessed using the SLE Responder Index 4 (SRI‐4).	None	The serum biomarker assessments done in these trials indicate that an IL-12 blockade plays a very important role in the M.O.A of ustekinumab treatment in SLE.
4.	Cheng LE, Amoura Z et al	phase Ib, randomized, double‐blind, placebo‐controlled study	Patients were adults ages 18–65 years with a diagnosis of SLE for ≥6 months as defined by the American College of Rheumatology criteria	none	210 mg AMG 557 or equivalent placebo subcutaneously once weekly for three weeks, followed by ten additional doses of AMG 557 or placebo every other week	Treatment response based on the Lupus Arthritis Responder Index (LARI)	None	Evidence of improvement was observed with AMG 557 administration as reflected by changes in the tender and swollen joint counts from the baseline.
5.	Clowse ME, Wallace DJ et al [5]	Phase III multicenter randomized, double‐blind, placebo‐controlled trials	Eligible patients were age ≥18 years and had a diagnosis of moderately to severely active SLE that fulfilled ≥4 of the 11 American College of Rheumatology (A.C.R.) revised criteria for SLE 10 (if patients were positive for a neurologic disorder, the diagnosis had to meet ≥5 of 11 A.C.R. criteria). All patients had, at a minimum, disease activity in the musculoskeletal, mucocutaneous, or cardiorespiratory body systems, as defined by the 2004 version of the BILAG index (BILAG‐2004.	Patients with severe lupus nephritis or severe neuropsychiatric SLE at screening	Placebo, epratuzumab 600 mg every week, or epratuzumab 1,200 mg every other week. Infusions were delivered over a 4‐week dosing period at the beginning of each 12‐week treatment cycle	The responder rate at week 48, according to the BICLA composite endpoint	None	Treatment of patients with moderately to severely active SLE with epratuzumab led to reductions in the levels of CD22, with the number of B cells in the peripheral blood decreasing by ∼30–40% and IgM levels reducing by 20%. However, treatment with epratuzumab in conjunction with standard therapy did not improve efficacy outcomes at week 48 compared to treatment with placebo in conjunction with standard treatment.
6.	Doria A, Bass D, et al [6]	Phase III, double-blind study	patients were ≥ 18 years of age with a diagnosis of SLE classified according to the American College of Rheumatology criteria,^13^ a Safety of Estrogens in Lupus Erythematosus National Assessment-Systemic Lupus Erythematosus Disease Activity Index (SELENA-SLEDAI) score ≥ 8 and were antinuclear antibody and anti-double-stranded D.N.A. (dsDNA) positive	Patients with severe lupus kidney disease or severe central nervous system lupus	Belimumab 200 mg sc weekly	Efficacy was evaluated by SRI4 at week 24 and is defined as a ≥4-point reduction from baseline in SELENA-SLEDAI score, no worsening (an increase of	none	Belimumab SC was well tolerated, and efficacy was maintained during the extension phase of this study. The safety profile of Belimumab S.C. is consistent with that of previous experience with Belimumab.
7.	Furie R, Petri M et al [7]	Multicenter, randomized, controlled, phase 3 trial						Belimumab plus S.O.C. significantly improved S.R.I. response rate, reduced SLE. disease activity, reduced severe flares, and was generally well-tolerated in SLE. Belimumab plus S.O.C. significantly improved S.R.I. response rate, reduced SLE disease activity, and reduced severe flares well-tolerated in SLE. Belimumab plus S.O.C. significantly improved S.R.I. response rate, reduced SLE.disease activity, and reduced severe flares well-tolerated in SLE. Belimumab plus S.O.C. significantly improved S.R.I. response rate, reduced SLE disease activity, and reduced severe flares well-tolerated in SLE. Belimumab plus S.O.C. significantly improved S.R.I. response rate, reduced SLE disease activity, and reduced severe flares well-tolerated in SLE.
8.	Furie R, Khamashta M, et al [8]	phase 3, multicenter, randomized, double-blind, placebo-controlled trial,	1) age ≥ 18 years; 2) a diagnosis of SLE. according to the revised American College of Rheumatology criteria; 3) active disease (SELENA-SLEDAI score ≥ 6) at screening; and 4) seropositivity as defined by two positive ANA or anti-dsDNA test results (ANA titers ≥ 1:80 and anti-dsDNA antibodies ≥ 30 I.U./mL), of which ≥ 1 test result had to be obtained during screening. The study entry criteria were identical to those in BLISS-52	Intercurrent illness, severe active lupus nephritis, severe central nervous system manifestations, and pregnancy. Prior treatment with a B-cell–targeted therapy was exclusionary, as was any investigational biologic agent within one year of screening or investigational nonbiologic agent within 60 days.	Belimumab 1 and 10 mg/kg plus SOC	S.R.I. response rate at week 52. An S.R.I. response was defined as a ≥ 4-point reduction in SELENA-SLEDAI score, no new BILAG A organ domain score, and no more than one new BILAG B score	none	Belimumab plus S.O.C. significantly improved S.R.I. response rate, reduced SLE disease activity and severe flares, and was generally well-tolerated in SLE. Belimumab plus S.O.C. improved S.R.I. response rate, reduced SLE. disease activity and intense flares, and were generally well-tolerated in SLE. Belimumab plus S.O.C. significantly improved S.R.I. response rate, reduced SLE disease activity and severe flares, and were generally well-tolerated in SLE.
9.	Zhang F, Bae SC, et al [9]	Phase III, randomized, placebo-controlled study	≥18 years of age who had a clinical diagnosis of SLE. according to the American College of Rheumatology classification criteria and clinically active disease, defined as a SELENA-SLEDAI score ≥8 at screening.	SLE patients who had severe lupus kidney disease or active nephritis that needed acute therapy within 90 days before baseline, or central nervous system (C.N.S.) lupus requiring treatment within 60 days before baseline, and those requiring new SLE medications.	Belimumab 10 mg/kg or placebo, plus SoC	SLE Responder Index (S.R.I.) 4 response rate at Week 52	The percentage of patients with ≥4 point reduction in Safety of Oestrogens in Lupus Erythematosus National Assessment-SLE Disease Activity Index (SELENA-SLEDAI), SRI7, time to first severe flare, and the number of days prednisone (or equivalent) dose ≤7.5 mg/day and reduced by 50% from baseline. Safety was assessed.	In patients with SLE from North-East Asia, Belimumab significantly improved disease activity decreasing the use of prednisone.
10.	Petri M, Wallace DJ, Spindler A, et al [10]	Phase I randomized controlled dose-escalation study.	≥18 years with moderate-to-severe SLE were enrolled in the study. All study participants were required to meet the 4 American College of Rheumatology revised classification criteria for SLE, have a Safety of Estrogens in Lupus Erythematosus National Assessment (SELENA) version of the SLE Disease Activity Index (SLEDAI) score of ≥6 or 1 system with a British Isles Lupus Assessment Group (BILAG) score of A or two systems with a BILAG score of B at screening, and have a positive antinuclear antibody test (≥1:80 serum dilution) at or before the screening.	Acute illness (other than SLE.) or infection; history of or current severe viral or tuberculosis infection, primary immunodeficiency, or cancer; herpes zoster infection within the past three months; abnormal blood test results at screening; recent high (>20 mg/day) or fluctuating doses of oral corticosteroids, antimalarials, or immunosuppressants; B cell–depleting therapies within the past 12 months, treatment with leflunomide in the past six months, or any other biologic agent in the past 30 days; treatment with sifalimumab in the past four months; or detectable anti-sifalimumab antibodies at screening.	IV sifalimumab (0.3, 1.0, 3.0, or 10.0 mg/kg)	Safety and tolerability of sifalimumab. The investigator recorded adverse events (A.E.s) and serious A.E.s (S.A.E.s) and their severity, outcome, and any relationship to the study medication.	Concentrations of sifalimumab in serum samples	The observed safety/tolerability and clinical activity profile of sifalimumab support its continued clinical development for SLE.

Future thrust

The following aspects of belimumab that are yet to be explored are the fact that the efficacy of belimumab as monotherapy is still to be proved, the safety and efficacy of Belimumab in lupus nephritis patients have not been established yet, the risk of developing neuropsychiatric manifestations of belimumab is a concern, especially in patients with CNS lupus, the safety and efficacy of belimumab in children and pregnant patients is yet to be established, the long-term safety of belimumab is still not established, and clinical and economic studies are to be done yet to know its feasibility.

## Conclusions

Based on a systematic review of the literature and clinical trials on the role and effect of belimumab on SLE, we can confidently say that belimumab appears to be efficacious and generally well-tolerated in the treatment of SLE as compared to other drugs. The long-term use of belimumab combined with standard therapy showed a low incidence of organ damage; patients who had a high risk for organ damage had a lower incidence of organ damage after initiating the treatment. Patients who test for antinuclear antibody or anti-dsDNA-positive SLE with moderate symptoms in the skin and musculoskeletal systems despite immunosuppressants are treated with belimumab as an adjunct therapy. Patients with severe lupus nephritis or active CNS lupus cannot be treated with belimumab. Belimumab is effective in most races, as a clinical trial done in North-East Asia showed improvement of SLE symptoms and decreased dependence on prednisone. Belimumab also decreased disease activity and severe flares. Finally, belimumab had greater efficacy in children.
